# When to shunt and when to aspirate? Long-term outcomes from a large stereotactic cohort of cystic craniopharyngiomas

**DOI:** 10.1016/j.bas.2026.106265

**Published:** 2026-08-07

**Authors:** Biyan Nathanael Harapan, Antonia Clarissa Wehn, Sabrina V. Kirchleitner, Moritz Ueberschaer, Mathias Kunz, Jun Thorsteinsdottir, Florian Ringel, Michael Schmutzer-Sondergeld

**Affiliations:** a Department of Neurosurgery, LMU University Hospital, LMU Medizin, Ludwig-Maximilians-Universität München, Marchioninistraße 15, Munich, 81377, Germany; b Department of Neurosurgery, University Hospital Marburg, University of Marburg, Baldingerstraße 1, Marburg, 35043, Germany; c Department of Neurosurgery, University Hospital Salzburg, Paracelsus Medical University, Salzburg, Austria

**Keywords:** Craniopharyngioma, Cystic craniopharyngioma, Stereotactic neurosurgery, Internal shunt, Cyst shunting, Stereotaxy

## Abstract

**Introduction:**

Stereotactic surgery of predominantly cystic craniopharyngiomas (CC) is a minimally invasive alternative to microsurgical or endoscopic resection. Both cyst aspiration (CA) and internal cyst shunting (CS) have been proposed as stereotactic treatment strategies, yet comparative data remain limited.

**Research question:**

Do CA and CS differ in clinical, radiological, visual, endocrine, and recurrence outcomes in predominantly cystic craniopharyngiomas?

**Material and methods:**

A retrospective cohort of patients treated between 2000 and 2025 was analyzed. Volumetric MRI was performed preoperatively and at ≤6 weeks, 4–6 months, and 9–12 months postoperatively. Clinical, radiological, visual, endocrine, and recurrence outcomes were evaluated using uni- and multivariate analyses.

**Results:**

A total of 42 patients were included (15 CA, 27 CS). Baseline characteristics were largely comparable, although optic nerve sheath dilation was more frequent in the CS group. Preoperative cyst volumes were similar (14.2 ± 10.3 cm^3^ vs. 16.5 ± 9.7 cm^3^; p = 0.5), as were final postoperative volumes (3.2 ± 3.4 cm^3^ vs. 4.2 ± 5.9 cm^3^; p = 0.6). Both techniques achieved significant and durable cyst reduction (all p < 0.001). Visual outcomes favored CS (85.2% vs. 53.3% without deficit; p = 0.02), whereas corticotropic (p = 0.02), thyreotropic insufficiency, and diabetes insipidus (both p = 0.03) were more frequent. Recurrence correlated with preoperative cyst volume (p = 0.008), independent of treatment modality.

**Discussion and conclusion:**

Both techniques provide effective cyst decompression. CS may improve visual outcomes but appears associated with higher endocrine risk. Findings support individualized treatment selection and warrant prospective validation.

## Highlights

• Largest stereotactic cohort comparing cyst aspiration (CA) and cyst shunting (CS) in predominantly cystic craniopharyngiomas.
• Cyst aspiration and cyst shunting achieved durable decompression.
• Cyst shunting was associated with superior postoperative visual recovery.
• Endocrinological deficits occurred more frequently after cyst shunting.
• Preoperative cyst volume predicted recurrence independent of treatment modality.

## 1 Introduction

Predominantly cystic craniopharyngiomas (CC) represent a distinct subgroup of craniopharyngiomas characterized by predominant cyst formation and a complex clinical course, for which optimal management remains debated (Calvanese et al., 2025; Bianchi et al., 2022; Sato et al., 2025; Chen and Sun, 2025). Despite their benign histology, these lesions frequently arise in the sellar–suprasellar region and in close proximity to critical neurovascular structures, including the optic apparatus and hypothalamic–pituitary axis. As a result, both the disease itself and its treatment are associated with substantial risks of visual and endocrinological morbidity. While microsurgical and endoscopic approaches have traditionally been considered standard treatment options (Rachinger et al., 2017; Chen et al., 2025), particularly in symptomatic or progressive cases, their invasiveness and potential for long-term functional impairment have driven increasing interest in minimally invasive alternatives.

**Fig. 1:**
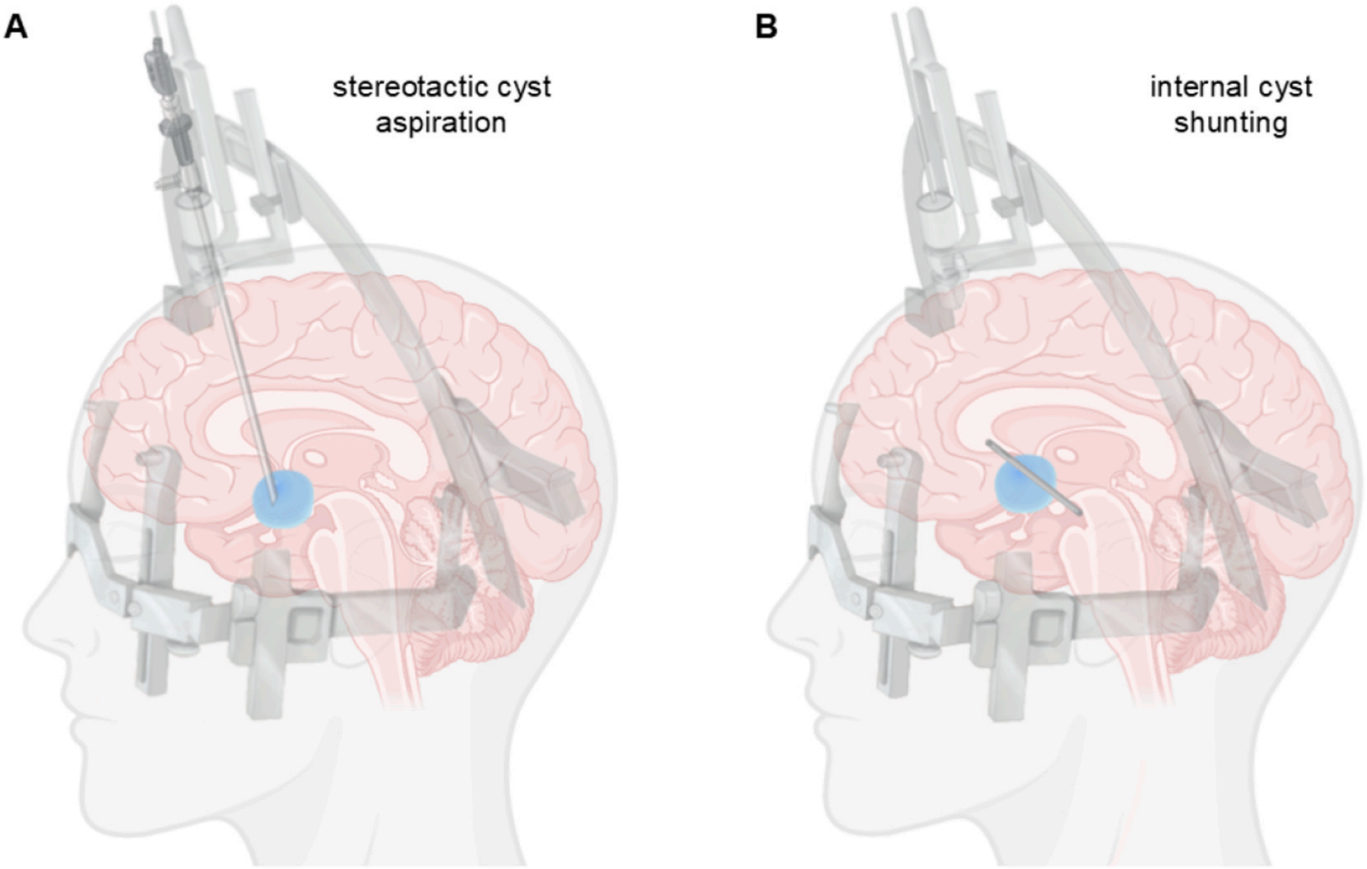
Schematic representation of the stereotactic treatment modalities applied in cystic craniopharyngiomas (CC). The illustration depicts stereotactic cyst aspiration (CA), involving targeted one-time decompression of the cyst cavity (A), and stereotactic internal cyst shunting (CS), establishing continuous drainage into the ventricular system (B).

Stereotactic procedures, such as cyst aspiration (CA) and stereotactic internal cyst shunting (CS), have emerged as promising strategies that allow targeted decompression with reduced surgical morbidity (Fig. 1). (Schmutzer-Sondergeld et al., 2024)

These approaches are particularly attractive in cyst-dominant lesions, where symptomatology is largely driven by mass effect and fluid accumulation rather than solid tumor burden (Schmutzer-Sondergeld et al., 2025). A previous study has shown that stereotactic drainage of cystic craniopharyngiomas can achieve significant and sustained volume reduction, with favorable clinical and endocrinological outcomes compared to more invasive surgical strategies (Rachinger et al., 2017). In particular, internal shunting offers the theoretical advantage of continuous cyst decompression, potentially mitigating rapid reaccumulation driven by secretory epithelial activity.

Importantly, data directly comparing CA and CS in predominantly cystic craniopharyngiomas remain limited. Existing stereotactic drainage and shunting techniques have been described predominantly in the context of arachnoid cysts (Gangemi et al., 2011; Hayes et al., 2019), whereas reports focusing specifically on CC are extremely rare and largely confined to small, heterogeneous cohorts. As a result, the relative advantages, recurrence rate, and outcome profiles of these two stereotactic strategies – particularly regarding symptom improvement, durability of decompression, recurrence patterns, visual outcomes, and endocrine function – remain insufficiently characterized in this specific cyst entity.

Given these limitations, the present study analyzes, to our knowledge, the largest cohort to date of newly diagnosed predominantly cystic craniopharyngiomas treated with stereotactic techniques, directly comparing cyst aspiration and internal cyst shunting with regard to clinical, radiological, and functional outcomes. In addition to detailed assessment of symptom relief and volumetric response, disease-specific parameters, including the Craniopharyngioma Clinical Status Scale (CCSS), were evaluated to provide a comprehensive characterization of treatment effects and to support a more differentiated, evidence-based approach to stereotactic management in this challenging patient population.

## 2 Material and methods

### 2.1 Study design and objectives

This retrospective, observational cohort study investigated the clinical, radiological, and surgical characteristics of patients who underwent stereotactic treatment for predominantly cystic craniopharyngiomas (CC) at the Department of Neurosurgery, LMU University Hospital, Munich, between January 2000 and May 2025. The primary objective was to compare outcomes between two stereotactic treatment modalities: stereotactic cyst aspiration (CA) and stereotactic internal shunt placement (CS). Secondary objectives included analysis of volumetric cyst changes across multiple postoperative time points, evaluation of symptom improvement, visual and endocrine outcomes, recurrence patterns, and assessment of factors associated with recurrence and treatment modality.

### 2.2 Patient selection and inclusion criteria

All patients receiving stereotactic intervention for CC within the defined study period were identified through a systematic review of the institutional stereotactic surgery database. Inclusion criteria were as follows: (1) diagnosis of a predominantly cystic craniopharyngioma based on preoperative MRI; (2) stereotactic treatment with either CA or CS performed at our institution; (3) availability of complete preoperative and postoperative clinical and radiological data; and (4) minimum follow-up of one postoperative imaging time point. Patients with incomplete imaging datasets, prior surgical intervention, or insufficient documentation were excluded.

### 2.3 Treatment protocol

Stereotactic management consisted of either the placement of an internal shunt catheter (CS), creating a permanent conduit between the cyst cavity and the ventricular system, or stereotactic cyst aspiration (CA), depending on the individual anatomical configuration and spatial relationship of the cystic craniopharyngioma (CC). Surgical planning (iPlan Stereotaxy; Brainlab, Munich, Germany) was performed using a stereotactically registered contrast-enhanced computed tomography (CT) scan (slice thickness 0.6 mm), which was rigidly fused with preoperative MRI sequences, including T1-weighted, T2-weighted/CISS, and contrast-enhanced MRA datasets.

For CS, a 1.3-mm ventricular catheter (Becker EDMS Ventricular Catheter; Medtronic Inc., Dublin, Ireland) was implanted stereotactically through a 2-mm burr hole. Additional side perforations were manually created along the distal catheter segment to optimize fluid drainage. The shunt trajectory was advanced through the anterior horn of the lateral ventricle and the foramen of Monro, traversing the cyst and terminating in the prepontine cistern, enabling continuous drainage into the ventricular system.

The catheter was secured extracranially using a titanium hemoclip (Titanium Ligation Clip, 150 mm; B. Braun, Melsungen, Germany) positioned orthogonally on the calvarial surface to prevent intraparenchymal migration. A collagen-based fibrin sealant patch (TachoSil®, Takeda Pharmaceuticals, Konstanz, Germany) was applied over the burr hole for watertight closure and added fixation (Schmutzer-Sondergeld et al., 2024; Schmutzer-Sondergeld et al., 2024; Niedermeyer et al., 2024/01; Schmutzer-Sondergeld et al., 2025; Lenski et al., 2019; Thorsteinsdottir et al., 2025; Kirchleitner et al., 2025). For CA, trajectories analogous to those used for CS were selected, with the target located within the cyst to allow aspiration of cyst contents. The choice between CA and CS was not randomized but was determined on a case-by-case basis by an experienced neurosurgical team. Treatment decisions were guided by a combination of anatomical, radiological, and clinical factors, including cyst size and configuration/morphology, proximity to the optic apparatus and hypothalamic–pituitary axis, presence of hydrocephalus or radiological signs of elevated intracranial pressure (e.g., optic nerve sheath dilation), as well as the severity of visual and endocrinological deficits.

### 2.4 Data collection and clinical parameters

Clinical, radiological, and surgical data were retrospectively extracted from the institutional electronic medical records, stereotactic planning documentation, and radiology archives. Demographic parameters included age at surgery, sex, stereotactic technique applied (CA or CS), date of surgical intervention, and duration of follow-up. Procedural details such as the planned stereotactic trajectory, burr-hole location, use of catheter implantation in CS, and procedure-related complications including hemorrhage or infection were systematically documented.

Radiological parameters included the presence of hydrocephalus or ventricular enlargement, prominent optic nerve sheaths, intralesional hemorrhage, and cyst rupture. The Craniopharyngioma Clinical Status Scale (CSSS) (Elliott et al., 2010) was employed to assess outcome across 5 axes, including neurological examination, visual status, pituitary function, hypothalamic dysfunction, and educational/occupational status (N-, V-, P-, H-, E-CCSS). Functional status was evaluated using the modified Rankin Scale (mRS) (Banks and Marotta, 2007) and Karnofsky Performance Status (KPS) (Péus et al., 2013; Mor et al., 1984).

Symptom burden was assessed pre- and postoperatively, including headache, mild and severe visual impairment, vertigo, epileptic seizures, and cognitive or mnestic disturbances. Detailed endocrinological evaluation was performed pre- and postoperatively, covering corticotropic, somatotropic, thyreotropic, and gonadotropic axes, as well as the presence of diabetes insipidus. Visual and perimetrical function were documented based on ophthalmological assessment (e.g., mild/severe deficits, partial anopsia, hemianopsia). Deficits in visual acuity were classified as no deficit (visual acuity 1.0), mild deficit (visual acuity 0.5-0.9) and severe deficit (visual acuity 0-0.4), and visual field deficits as no deficit, partial anopsia or complete hemianopsia.

A progression was defined as either a postoperative increase in cyst volume greater than 25% or a worsening of symptoms related to the remaining cyst. The time to recurrence (TTR) was recorded in days. Follow-up duration extended to the last available clinical or radiological evaluation.

### 2.5 Imaging and volumetric analysis

**Fig. 2:**
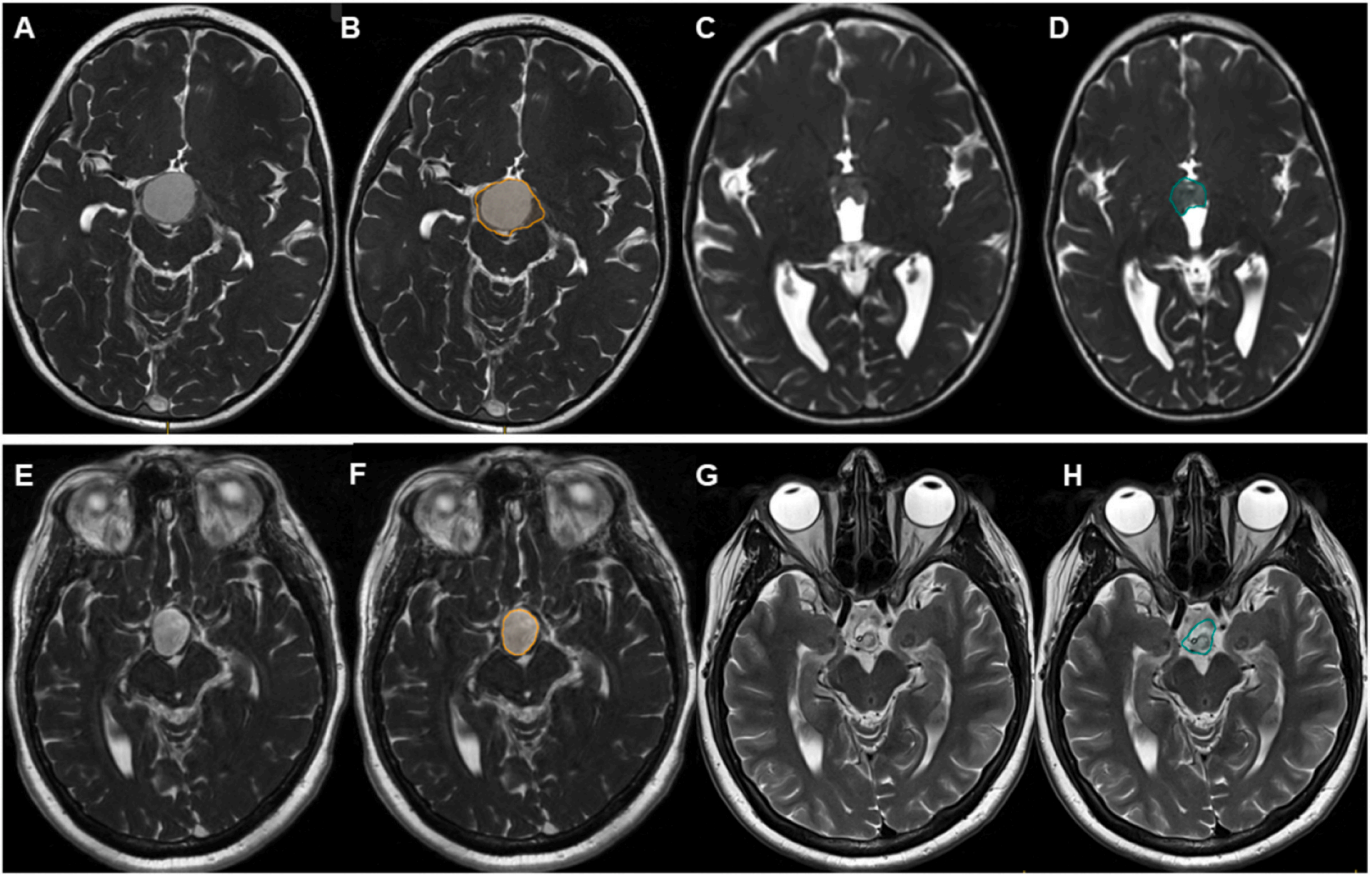
Representative pre- and postoperative CISS MRI scans of predominantly cystic craniopharyngiomas. A–D: Case treated with stereotactic cyst aspiration showing preoperative imaging (A) with corresponding volumetric segmentation (B) and postoperative cyst reduction (C) with segmentation (D). E–H: Case treated with stereotactic internal shunting showing preoperative imaging (E) with segmentation (F) and postoperative cyst reduction (G) with segmentation (H).

Radiological morphological diagnosis of cystic craniopharyngiomas was established by an experienced interdisciplinary team based on the characteristic radiological appearance. MRI criteria included a predominantly cystic sellar/suprasellar lesion with typical morphology, well-defined margins, and a thin enhancing cyst wall following gadolinium administration. The cystic component typically demonstrated variable T1-weighted signal intensity ranging from iso- to hyperintense relative to gray matter, while appearing predominantly hyperintense on T2-weighted sequences. Patients with atypical imaging findings or diagnostic uncertainty were excluded from the present analysis. Cyst volumetry was performed on preoperative MRI and at three standardized postoperative intervals: up to six weeks postoperatively, four to six months postoperatively, and nine to twelve months postoperatively. All MRI scans were obtained using 1.5- or 3.0-T scanners (Magnetom Symphony or Magnetom Skyra, Siemens Healthineers, Erlangen, Germany), following a uniform protocol consisting of axial and coronal T1-weighted sequences before and after intravenous gadolinium administration (0.1 mmol/kg), along with T2-weighted and FLAIR sequences. Volumetric measurements were carried out using semiautomated segmentation software (SmartBrush®, Elements®, Brainlab AG, Munich, Germany) (Harapan et al., 2026a, 2026b). Cyst boundaries were manually traced on T2-weighted axial images, with confirmation in sagittal and coronal planes to ensure spatial accuracy. Postoperative segmentation was performed using identical imaging parameters (Fig. 2). Absolute and relative volume reductions were calculated by comparing postoperative cyst volumes to preoperative baseline measurements.

### 2.6 Statistical analysis

Statistical evaluation was conducted using GraphPad Prism version 10.0 (GraphPad Software, San Diego, CA, USA). Continuous variables were expressed as mean ± standard deviation and compared using unpaired or paired t-tests. Categorical variables were evaluated using chi-square or Fisher's exact tests. Longitudinal volumetric changes were analyzed with repeated-measures testing. Logistic regression identified predictors of recurrence and factors associated with treatment modality. Variables with p < 0.05 in univariate analysis entered multivariate models. Statistical significance was defined as p < 0.05.

### 2.7 Ethical considerations

The study was conducted in accordance with the Declaration of Helsinki (2013 revision). Ethical approval was obtained from the Ethics Committee of the LMU Munich (project number 19-798). All data were pseudonymized prior to analysis and stored securely on institutional servers. Clinical and imaging data were collected as part of routine care and evaluated retrospectively for research purposes.

## 3 Results

### 3.1 Patient characteristics

Among the 42 CC patients, 15 (35.7%) underwent CA and 27 (64.3%) underwent CS. The cohort included 18 males (42.9%) and 24 females (57.1%), with similar age distributions (CA: 25.2 ± 25.9 years vs. CS: 34.1 ± 23.0 years; age range: 1.8–84.6 years; p = 0.3). Preoperative functional status, including KPS and mRS, as well as ventricular enlargement, were comparable between groups. In contrast, prominent optic nerve sheath dilation – serving as a radiological surrogate of increased intracranial pressure – was more frequently observed in CS patients (37.0% vs. 6.7%, p = 0.03). Additionally, preoperative BMI was also significantly higher in the CS group (26.2 ± 5.7 vs. 19.8 ± 6.7 kg/m^2^, p = 0.009) (Table 1).

**Table 1:** Baseline demographic and clinical characteristics of patients with predominantly cystic craniopharyngiomas treated with cyst aspiration (CA) or stereotactic cyst shunting (CS).

Parameters	Stereotactic cyst aspiration (CA)	Stereotactic cyst shunting (CS)	*p value*
Patient characteristics			
**Total, n (%)**	15 (35.7)	27 (64.3)	
**Sex, n (%)**			
male	6 (40.0)	12 (44.4)	0.8
female	9 (60.0)	15 (55.6)	
**Age, years**	25.2 ± 25.9	34.1 ± 23.0	0.3
**Karnofsky Performance Status (KPS), %**	89.3 ± 10.3	91.2 ± 7.3	0.5
**Hemorrhage, n (%)**	0/15 (0)	0/27 (0)	0.99
**Rupture, n (%)**	0/15 (0)	0/27 (0)	0.99
**Expanded ventricular system, n (%)**			
preoperative	5/15 (33.3)	11/27 (40.7)	0.6
postoperative	3/15 (20.0)	2/27 (7.4)	0.2
**Prominent optic nerve sheaths, n (%)**			
preoperative	1/15 (6.7)	10/27 (37.0)	**0.03**
postoperative	0/15 (0)	0/27 (0)	0.99
**Volume (cm^3^)**			
preoperative	14.2 ± 10.3	16.5 ± 9.7	0.5
postoperative	3.2 ± 3.4	4.2 ± 5.9	0.6
**Modified Rankin Scale (mRS)**			
preoperative	1.5 ± 0.7	1.5 ± 0.6	0.9
postoperative	1.3 ± 0.6	1.2 ± 0.5	0.3
**BMI (kg/m^2^)**			
preoperative	19.8 ± 6.7	26.2 ± 5.7	**0.009**
postoperative	23.0 ± 8.1	27.8 ± 7.6	0.1
**Follow-Up, months**	44.0 ± 66.6	59.9 ± 58.7	0.4
**Time to second surgery (recurrence), days**	341.0 ± 202.0	427.0 ± 331.6	0.7
**Recurrences, n (%)**	3/15 (20.0)	10/27 (37.0)	0.3
**Complications, n (%)**	1/15 (6.7)	0/27 (0)	0.4
**Total number of surgeries, n (%)**	1.3 ± 0.6	1.6 ± 0.8	0.2
**Craniopharnygioma Clinical Status**			
**Scale (CCSS)**			
**N-CCSS**			
preoperative	1.5 ± 0.5	1.8 ± 0.4	0.08
postoperative	1.4 ± 0.5	1.1 ± 0.3	**0.01**
**V-CCSS**			
preoperative	1.9 ± 0.9	1.6 ± 0.8	0.2
postoperative	1.8 ± 0.9	1.4 ± 0.8	0.2
**P-CCSS**			
preoperative	1.6 ± 0.8	1.5 ± 0.9	0.8
postoperative	1.8 ± 0.9	2.4 ± 1.1	0.1
**H-CCSS**			
preoperative	1.1 ± 0.3	1.2 ± 0.6	0.3
postoperative	1.1 ± 0.5	1.4 ± 0.8	0.2
**E-CCSS**			
preoperative	1.3 ± 0.5	1.4 ± 0.5	0.7
postoperative	1.7 ± 0.7	1.6 ± 0.6	0.4

Regarding anatomical distribution, no purely intrasellar lesions were included. Twenty-five tumors were located within the sellar region with suprasellar extension (10 treated with CA and 15 with CS), 10 lesions were purely suprasellar without intraventricular extension (2 CA, 8 CS), and 7 lesions were suprasellar with extension into the third ventricle (3 CA, 4 CS). Exclusively intraventricular craniopharyngiomas were therefore not represented in this cohort.

### 3.2 Radiological outcomes

**Fig. 3:**
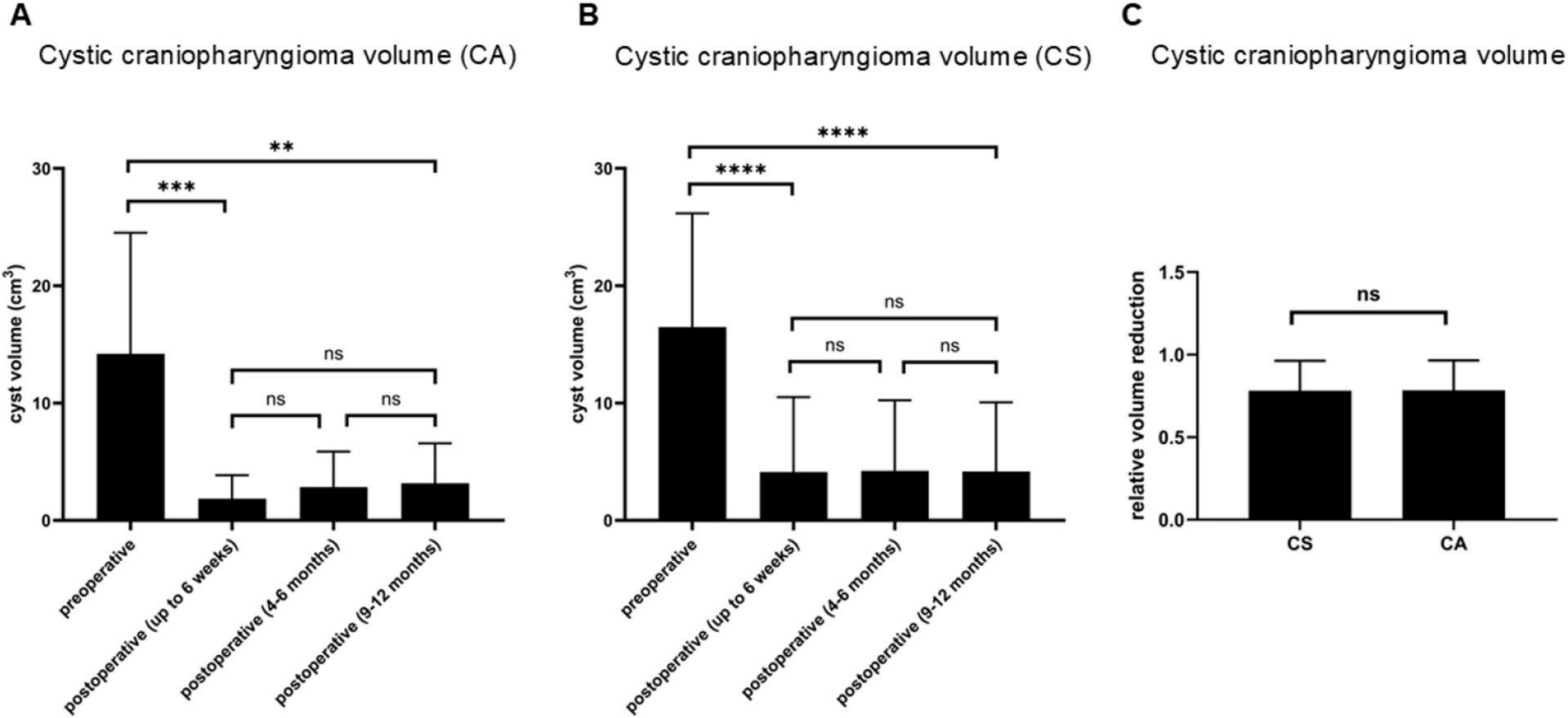
Volumetric analysis of cystic craniopharyngiomas treated with cyst aspiration (CA) or stereotactic internal shunting (CS). Bars represent mean cyst volumes with standard deviation (SD), with error bars indicating SD. CA (A) and CS (B) similarly achieved substantial and sustained volume reductions (****p < 0.0001; ***p = 0.0002; **p = 0.0008), with no significant difference in relative reduction between modalities (C).

Baseline cyst volumes did not differ significantly (CA: 14.2 ± 10.3 cm^3^ vs. CS: 16.5 ± 9.7 cm^3^; p = 0.5). Final postoperative volumes were also similar (CA: 3.2 ± 3.4 cm^3^ vs. CS: 4.2 ± 5.9 cm^3^; p = 0.6). Both CA and CS achieved substantial and durable cyst reduction, with significant decreases at all postoperative time points (all p < 0.001) (Fig. 3A and B). Absolute and relative reductions (calculated on a per-patient basis) were comparable (absolute: 10.3 ± 9.2 cm^3^ vs. 12.3 ± 6.8 cm^3^; p = 0.4; relative: both 0.8 ± 0.2; p = 0.9) (Fig. 3C).

### 3.3 Clinical, visual, and endocrinological outcomes

Headache was the most common presenting symptom (61.9%), followed by mild visual impairment (21.4%) and severe visual impairment (16.7%). Postoperatively, patients experienced significant improvement in headache (p < 0.0001) and mnestic symptoms (p = 0.02). Severe visual impairment also showed a postoperative reduction, approaching statistical significance (p = 0.08) (Table 2).

**Table 2:** Preoperative and postoperative symptom profiles in patients with cystic craniopharyngiomas undergoing cyst aspiration (CA) or stereotactic cyst shunting (CS).

	Preoperative n (%)	Postoperative n (%)	*p value*
***Symptoms***			
none	0/42 (0)	30/42 (71.4)	**<0.0001**
headache	26/42 (61.9)	1/42 (2.4)	**<0.0001**
mnestic disorder	5/42 (11.9)	0/42 (0)	**0.02**
mild visual impairment	9/42 (21.4)	9/42 (21.4)	0.99
severe visual impairment	7/42 (16.7)	2/42 (4.8)	0.08
paresis	0/42 (0)	0/42 (0)	0.99
sensory disturbance	0/42 (0)	0/42 (0)	0.99
epileptic seizures	0/42 (0)	0/42 (0)	0.99
vertigo	2/42 (4.8)	0/42 (0)	0.1
coordination disturbance	1/42 (2.4)	0/42 (0)	0.3
cranial nerve symptoms	0/42 (0)	0/42 (0)	0.99
growth disorder	3/42 (7.1)	2/42 (4.8)	0.6

Postoperatively, both treatment modalities resulted in meaningful clinical improvement; however, visual outcomes favored the stereotactic cyst shunt (CS) group. Specifically, 85.2% of CS patients showed no residual visual deficit, compared with 53.3% in the CA group (p = 0.02). The absence of postoperative severe visual deficits in the CS cohort (0%) contrasted with two cases of persisting severe deficits (13.3%) in the CA cohort, representing a trend toward a protective effect of shunting on visual deficiencies (p = 0.05) (Table 3). Endocrinological evaluation revealed a postoperative increase in hormone deficiencies primarily in CS patients, with significant rises in corticotropic insufficiency (p = 0.02), thyreotropic dysfunction (p = 0.03), and diabetes insipidus (p = 0.03). Notably, relative cyst volume reduction was comparable between patients who developed postoperative endocrinological deterioration (75.6%) and those with stable or improved hormonal function (79.0%), with no significant difference observed (p = 0.7). CA patients showed no significant postoperative increases in endocrine deficiencies (Table 3).

**Table 3:** Overview of preoperative and postoperative endocrinological, visual, and perimetrical outcomes in patients with cystic craniopharyngiomas treated with cyst aspiration (CA) or stereotactic cyst shunting (CS).

	Preoperative CA n (%)	Postoperative CA n (%)	*p value*	Preoperative CS n (%)	Postoperative CS n (%)	*p value*
**Endocrinological outcome**						
No deficiency	9/15 (60.0)	7/15 (46.7)	0.5	17/27 (63.0)	9/27 (33.3)	**0.03**
Anterior hypopituitarism	3/15 (20.0)	4/15 (26.7)	0.7	6/27 (22.2)	8/27 (29.6)	0.5
Posterior hypopituitarism	2/15 (13.3)	2/15 (13.3)	0.99	1/27 (3.7)	1/27 (3.7)	0.99
Panhypopituitarism	1/15 (6.7)	2/15 (13.3)	0.5	3/27 (11.1)	9/27 (36.0)	**0.04**
***Endocrinological deficiencies***						
Corticotropic	1/15 (6.7)	2/15 (13.3)	0.5	4/27 (16.0)	12/27 (48.0)	**0.02**
Somatotropic	2/15 (13.3)	2/15 (13.3)	0.99	5/27 (20.0)	8/27 (32.0)	0.3
Thyreotropic	2/15 (13.3)	4/15 (26.7)	0.3	4/27 (16.0)	11/27 (44.0)	**0.03**
Gonadotropic	1/15 (6.7)	2/15 (13.3)	0.5	3/27 (12.0)	8/27 (32.0)	0.09
Diabetes insipidus	2/15 (13.3)	4/15 (26.7)	0.3	4/27 (16.0)	11/27 (44.0)	**0.03**
***Visual outcome***						
No deficit	8/15 (53.3)	8/15 (53.3)	0.99	18/27 (66.7)	23/27 (85.2)	0.1
Mild deficit	5/15 (33.3)	5/15 (33.3)	0.99	4/27 (14.8)	4/27 (14.8)	0.99
Severe deficit	2/15 (13.3)	2/15 (13.3)	0.99	5/27 (18.5)	0/27 (0)	**0.02**
***Perimetrical outcome***						
No deficit	5/15 (33.3)	4/15 (26.7)	0.7	8/27 (29.6)	23/27 (85.2)	**<0.0001**
Partial anopsia	7/15 (46.7)	9/15 (60.0)	0.5	12/27 (44.4)	4/27 (14.8)	**0.02**
Hemianopsia	3/15 (20.0)	2/15 (13.3)	0.6	7/27 (25.9)	0/27 (0)	**0.005**

Adjuvant radiotherapy was administered in a minority of patients following stereotactic cyst decompression. In the CS group, four patients received subsequent radiotherapy at a mean interval of 24.1 ± 10.0 months after the stereotactic procedure. In the CA group, one patient underwent radiotherapy 7 months after treatment. Thus, most patients achieved adequate disease control following stereotactic decompression alone and did not require immediate additional irradiation.

### 3.4 Recurrence, predictors of recurrence, and complications

**Fig. 4:**
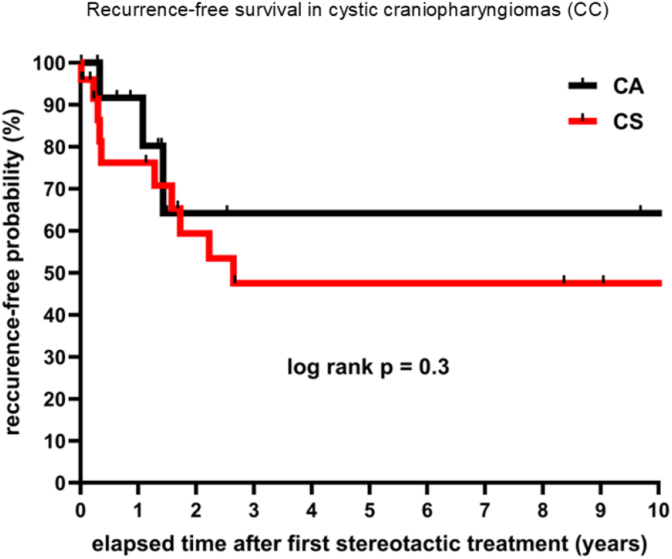
Kaplan–Meier curves depict time to recurrence (TTR) in patients with predominantly cystic craniopharyngiomas after cyst aspiration (CA) or stereotactic cyst shunting (CS). Curves illustrate recurrence-free survival for each treatment modality, with events defined as radiological cyst progression or symptomatic recurrence requiring further intervention.

Recurrence occurred in 3/15 CA patients (20.0%) and 10/27 CS patients (37.0%), without significant group differences (p = 0.3). Time to recurrence was comparable (Fig. 4). Recurrences following CS were managed with cyst aspiration in three cases, repeat internal shunting in four cases, and microsurgical intervention in three cases. In contrast, recurrences after CA were treated with internal shunting in two cases and microsurgical resection in one case.

Univariate analysis identified preoperative cyst volume (p = 0.008, OR 12.8) as the sole predictor of recurrence. Factors associated with the choice of surgical technique in univariate analysis (postoperative N-CCSS, cumulative vision, corticotropic dysfunction) did not remain significant in multivariate analysis.

One complication occurred following cyst aspiration, consisting of postoperative hemorrhage that was managed conservatively. The complication rate did not differ significantly between CA and CS (p = 0.4).

## 4 Discussion

The present study provides the most comprehensive stereotactic series to date directly comparing cyst aspiration (CA) and internal cyst shunting (CS) for predominantly cystic craniopharyngiomas (CC). Both techniques produced substantial and durable cyst decompression, with significant long-term radiological and clinical improvement. Our findings support stereotactic interventions as effective, low-morbidity strategies in anatomically delicate midline lesions where microsurgical or endoscopic approaches may carry higher risk.

As the present cohort consisted of patients undergoing stereotactic treatment for predominantly cystic craniopharyngiomas, the primary objective of the procedure was minimally invasive cyst decompression rather than tissue sampling. Therefore, solid tumor tissue was generally not available for histopathological examination. Aspiration typically yielded only the characteristic cyst fluid associated with cystic craniopharyngiomas, which is usually insufficient for meaningful histological evaluation. Cytological analysis of the aspirated fluid was not routinely performed, as it was not expected to influence diagnostic assessment or subsequent clinical management.

Cyst aspiration and internal shunting achieved comparable volumetric reduction, while clinical outcomes differed in selected functional domains. Recurrence was not independently associated with the choice of stereotactic technique but was primarily driven by lesion-specific factors, particularly preoperative cyst volume. These findings underscore the importance of intrinsic tumor biology, including secretory epithelial activity, in determining cyst behavior and recurrence risk. Given the propensity of craniopharyngioma cysts for reaccumulation, cyst aspiration may provide effective short-term decompression in selected cases, whereas internal shunting may be advantageous in patients at higher risk for rapid cyst re-expansion or requiring sustained decompression. The higher frequency of prominent preoperative optic nerve sheath dilation among patients selected for shunting suggests that this radiological marker may serve as a surrogate for CSF stasis and guide treatment allocation (Salih et al., 2024; Jayanth et al., 2021).

Both stereotactic procedures achieved significant and sustained cyst shrinkage; however, clinical outcomes diverged in domains relevant for long-term functional preservation. Internal shunting was associated with superior postoperative visual recovery and a lower proportion of persistent or severe visual deficits. This effect likely reflects the pathophysiological characteristics of craniopharyngioma cysts, which frequently reaccumulate viscous or proteinaceous fluid due to secretory epithelial activity (Massimi et al., 2017). A single aspiration may provide only transient decompression, whereas continuous drainage via an internal shunt might maintain stable pressure relief on the optic apparatus, thereby promoting more complete visual pathway recovery. These findings align with previous reports describing favorable ophthalmological outcomes after stereotactic cysto-ventricular catheter placement. In particular, the study by Steiert et al. demonstrated that catheter-based long-term cyst drainage achieved cyst volume reductions exceeding 60% and improved visual acuity in 90% of affected patients, without postoperative visual deterioration (Steiert et al., 2021). Their results reinforce the notion that durable decompression via internal drainage may provide meaningful visual benefit in selected patients with chiasmal compression. Our findings extend the existing evidence by directly comparing CS and CA, suggesting a potential advantage of CS with regard to postoperative visual normalization. Despite comparable absolute and relative cyst volume reduction, the superior visual outcomes observed after CS suggest that visual recovery is more dependent on the durability and temporal stability of decompression than on static postoperative cyst size alone. Continuous cyst–ventricular drainage may prevent subtle pressure fluctuations or early re-expansion that are sufficient to impair recovery of the optic apparatus following aspiration. However, interpretation of the observed superiority of shunting in visual outcomes is limited by the non-randomized treatment allocation. Patients selected for shunting more frequently exhibited preoperative signs of elevated intracranial pressure and greater chiasmal compression, including optic nerve sheath dilation, indicating a relevant baseline imbalance. This suggests that individuals with more pronounced but potentially reversible visual impairment were preferentially treated with shunting, therefore predisposing this group to greater postoperative improvement regardless of technique. Consequently, the apparent visual benefit of shunting should be interpreted with caution, as it may partly reflect underlying differences in baseline disease severity rather than a purely treatment-specific effect.

An important mechanistic consideration pertains to long-term shunt durability. Craniopharyngioma cysts may frequently contain mucinous or cholesterol-rich debris (Ainiwan et al., 2022) capable of obstructing the small fenestrations and luminal pathways of internal shunts. Catheter obstruction with proteinaceous debris – well recognized in ventricular shunting in general – represents an additional potential complication, particularly relevant in cystic lesions with highly protein-rich fluid (Paff et al., 2018; Garcia-Bonilla et al., 2024; Harris and McAllister, 2012). Such microscopic occlusion may occur gradually, clinically manifesting as secondary deterioration in vision, renewed headache, or radiographic cyst re-expansion after an initially successful postoperative course. Generally, this phenomenon provides a plausible explanation for a subset of recurrences observed in shunt patients and highlights the broader principle that shunt malfunction – even in the absence of classic signs of raised intracranial pressure – may precipitate significant visual decline. This has been highlighted in other clinical contexts, such as ventriculoperitoneal shunting, where visual impairment has been reported as the primary manifestation of shunt failure despite minimal headache or ventricular enlargement, often resulting in irreversible deficits when diagnosis is delayed (Qiu et al., 2022). A similar mechanism has been reported in an individual case of cyst-peritoneal shunt malfunction, where delayed catheter obstruction resulted in progressive visual decline despite initially sufficient cyst decompression (Ono et al., 2022).

In our cohort, the visual advantage of shunting occurred alongside a higher incidence of postoperative endocrinopathies, particularly corticotropic and thyreotropic deficiencies and diabetes insipidus. Notably, internal CS was associated with a higher rate of postoperative panhypopituitarism compared with CA. This finding likely reflects the anatomical and procedural demands of shunt placement in the sellar–suprasellar region, where catheter trajectories necessarily traverse or closely approach the hypothalamic–pituitary axis, which is often already compromised by chronic compression or inflammation. In addition, gradual cyst collapse during continuous drainage may induce subtle traction or vascular alterations of the pituitary stalk, a dynamic process that may not be observed after single-time aspiration. Consequently, cyst shunting may be an appropriate option in patients with limited hypothalamic reserve or preexisting endocrinological deficits, in whom additional hormonal impairment is less clinically consequential, whereas cyst aspiration – associated with less endocrine morbidity – may be preferable in patients with preserved endocrinological function. Importantly, these findings should be interpreted in the context of baseline anatomical differences between the treatment groups. Patients undergoing internal shunting exhibited a significantly higher preoperative BMI, which may represent a surrogate marker of more advanced hypothalamic involvement. Since both stereotactic aspiration and internal shunting utilize essentially identical stereotactic trajectories, differences in endocrine outcome are unlikely to be explained solely by procedural access. Rather, pre-existing hypothalamic dysfunction and disease-related anatomical factors probably contributed substantially to the observed endocrine differences. Consistent with this interpretation, multivariate analysis did not identify treatment modality as an independent predictor of postoperative endocrine dysfunction.

Recurrence was significantly associated with preoperative cyst volume, likely reflecting increased secretory activity and greater metabolic potential of larger cyst walls. Furthermore, recurrence risk was not independently influenced by the choice of stereotactic technique, suggesting that patient-specific anatomical and biological factors may play a more decisive role than the intrinsic differences between cyst aspiration and internal shunting.

The Craniopharyngioma Clinical Status Scale (CCSS) allows standardized assessment of neurological (N), visual (V), endocrine/pituitary (P), hypothalamic (H), and educational/occupational (E) status (Elliott et al., 2010). In our cohort, pre-to post-operative scores generally improved or remained stable, with a significant difference between treatment groups only observed in the N-CCSS domain (Table 1). Interestingly, the differences in visual and endocrinological outcomes identified in more granular analyses were not reflected in CCSS scores. While this may suggest limited sensitivity of the scale for detecting domain-specific changes, it may alternatively indicate that these differences – although statistically significant – are of limited clinical magnitude and therefore not captured by a validated composite outcome measure.

Taken together, our findings may support a selective, criteria-based approach to stereotactic treatment. Aspiration represents an appropriate, low-morbidity option for most craniopharyngioma patients in whom endocrine preservation is prioritized and rapid fluid reaccumulation is not anticipated. Internal shunting is advantageous when durable decompression is required – particularly in cases with hydrocephalus, large-volume cysts, or significant preoperative visual impairment – but should be accompanied by awareness of the risk for delayed shunt obstruction and endocrine deterioration. Our findings should also be interpreted in the context of prior work from our institution. In particular, Rachinger et al. demonstrated that stereotactic drainage of cystic craniopharyngiomas is associated with substantially lower endocrinological morbidity compared with microsurgical resection, supporting a minimally invasive treatment paradigm (Rachinger et al., 2017). The present study extends these findings by directly comparing two stereotactic strategies – cyst aspiration and internal shunting – within this disease entity. While our data suggest a potential advantage of internal shunting with respect to visual outcomes, this finding should be considered in the context of the selection biases discussed above. Both stereotactic treatment strategies provided effective and durable cyst decompression, resulting in substantial improvement of compression-related symptoms. Although internal shunting showed favorable trends regarding visual recovery and recurrence rates, this observation must be interpreted cautiously due to the limited sample size. Therefore, the present data do not demonstrate clear superiority of one technique over the other, and the choice of treatment should be individualized based on clinical and anatomical considerations. Our results reinforce the role of stereotactic approaches as effective and function-preserving treatment options, while further refining patient selection and technique-specific considerations within this minimally invasive framework.

Successful stereotactic cyst drainage was achieved in the majority of patients in our cohort, with sustained clinical benefit and no immediate requirement for additional definitive treatment. This observation highlights that, in selected cases, predominantly cystic craniopharyngiomas may demonstrate prolonged radiological stability following minimally invasive decompression. Such findings support a staged management approach, in which stereotactic drainage provides effective symptom relief while potentially delaying or avoiding the morbidity associated with more invasive interventions, including microsurgical resection or radiotherapy. Nevertheless, careful long-term clinical and radiological surveillance remains essential to detect delayed cyst recurrence or progression requiring further treatment.

The role and timing of adjuvant radiotherapy after stereotactic cyst decompression remain individualized and depend on the subsequent clinical and radiological course. The primary objective of stereotactic treatment is rapid cyst volume reduction and alleviation of mass effect while minimizing treatment-related morbidity. Following successful decompression, a period of observation may be appropriate in selected patients, particularly when residual tumor burden is limited and clinical symptoms remain controlled. In our cohort, radiotherapy was reserved for patients with persistent or recurrent disease rather than applied routinely after stereotactic intervention, suggesting that cyst decompression alone may provide durable control in a substantial proportion of patients. Delaying radiotherapy until clinically indicated may avoid unnecessary treatment exposure and allows irradiation to be performed after reduction of cyst volume, potentially limiting radiation exposure to adjacent critical structures. However, given the small number of patients receiving adjuvant radiotherapy in this series, no definitive conclusions regarding the optimal timing or indications for irradiation can be drawn. Future studies with larger cohorts and longer follow-up are required to better define the role of radiotherapy within a multimodal treatment strategy for cystic craniopharyngiomas.

The growing importance of molecular characterization is reshaping the management of craniopharyngiomas, particularly the papillary subtype, in which BRAF V600E mutations represent a defining molecular alteration. Identification of this mutation has enabled the development of targeted BRAF and MEK inhibitor therapies, which have demonstrated promising clinical efficacy and may provide an effective treatment alternative in selected patients (Brastianos et al., 2023; Luo et al., 2025). Although routine tissue acquisition was not feasible in our minimally invasive stereotactic cohort, molecular profiling may become increasingly relevant for individualized treatment planning, particularly in patients with recurrent disease or when targeted therapy is being considered. As precision medicine continues to evolve, integration of molecular biomarkers into clinical decision-making may facilitate more personalized treatment strategies and optimize long-term outcomes.

This study has several limitations inherent to its retrospective, single-center design. Treatment allocation to cyst aspiration or internal shunting was not randomized but based on surgeon judgment and clinical presentation, introducing potential selection bias, particularly concerning preoperative visual status, ventricular enlargement, and cyst size. Here, optic nerve sheath dilation and endocrine dysfunction were not regarded as independent criteria favoring one stereotactic technique over the other, but rather as potential indicators of disease severity or chronic effects of the lesion. Although the cohort represents the largest stereotactic series for predominantly cystic craniopharyngiomas to date, subgroup analyses – especially for endocrine outcomes and recurrence – remain constrained by sample size, limiting statistical power to detect subtle intergroup differences. Imaging protocols evolved over the 25-year study period, with transitions in MRI hardware from 1.5T to 3T. Interscanner variability – particularly in T2-weighted signal characteristics – may introduce measurement error despite the use of standardized segmentation techniques. This effect is especially relevant for small postoperative residual volumes, where minor absolute differences may influence volumetric comparisons. Moreover, clinical assessments, including ophthalmological and endocrinological testing, were not uniformly available at all time points for all patients, potentially affecting the completeness of functional outcome comparisons. An additional limitation relates to baseline differences in endocrinological status between treatment groups. Patients selected for internal shunting had a significantly higher preoperative BMI, which may indicate pre-existing hypothalamic dysfunction. This imbalance suggests that the shunting cohort may have been more susceptible to postoperative endocrinological deterioration, independent of the surgical technique. Consequently, the higher rate of endocrine deficits observed after shunting should be interpreted with caution, as it may reflect underlying differences in baseline hypothalamic status rather than a purely procedure-related effect. Finally, follow-up duration – although not statistically significant – varied among patients and between treatment groups, with longer surveillance in the shunting cohort. This imbalance may have influenced recurrence detection – potentially increasing the likelihood of identifying recurrences after shunting while underestimating late events after aspiration – and may also affect the assessment of delayed shunt obstruction. Although time-to-event analyses partially account for this variability, differential follow-up remains a relevant confounder when interpreting recurrence patterns.

On the whole, our findings suggest the potential for a risk-adapted selection of stereotactic technique in predominantly cystic craniopharyngiomas. Cyst aspiration may be preferable in patients with moderate cyst volume and preserved endocrine function, whereas internal shunting may be advantageous in cases with larger lesions, relevant visual compromise, or signs of increased intracranial pressure, where sustained decompression appears critical. Non-randomized treatment allocation and limited sample size introduce potential bias, and the present data do not permit the establishment of definitive treatment algorithms. Rather, they highlight the need for prospective validation of risk-adapted strategies in this setting. Prospective, multicenter studies with standardized surgical indications and uniform follow-up protocols are required to validate these findings, while our present results may serve as a preliminary framework to help inform and harmonize surgical indication criteria in future investigations.

Although retrospective design and selection biases remain unavoidable limitations, our cohort size, standardized volumetric analysis, and direct comparison of two stereotactic modalities for cystic craniopharnygiomas provide meaningful insights into potential optimal treatment selection. Overall, the data support stereotactic techniques as effective primary interventions for predominantly cystic craniopharyngiomas and highlight the importance of individualized therapeutic decision-making based on CSF dynamics, visual status, cyst biology, and endocrinological status.

## 5 Conclusions

Stereotactic cyst aspiration (CA) and cyst shunting (CS) represent effective minimally invasive treatment options for predominantly cystic craniopharyngiomas (CC), achieving significant and sustained cyst volume reduction with overall clinical improvement. While radiological outcomes were comparable, differences in visual and endocrine outcomes were observed. The apparent visual benefit associated with cyst shunting should be interpreted with caution in light of baseline imbalances and potential selection bias, whereas the higher rate of endocrinological deterioration in the shunting group likely reflects both procedural factors and underlying hypothalamic dysfunction.

Recurrence was not independently associated with treatment modality but was primarily driven by preoperative cyst volume, underscoring the importance of lesion-specific characteristics and cyst biology. Given the retrospective design, limited cohort size, and heterogeneity in follow-up, these findings do not generally support the superiority of one technique over the other. Rather, they support an individualized treatment approach, in which cyst aspiration may be favored when endocrine preservation is prioritized, and cyst shunting considered in selected patients requiring more durable decompression. Prospective studies with standardized treatment algorithms are warranted to better define optimal patient selection and long-term outcomes.

## Declaration of competing interest

The authors declare that they have no known competing financial interests or personal relationships that could have appeared to influence the work reported in this paper.
